# A novel cold-adapted type I pullulanase of *Paenibacillus polymyxa* Nws-pp2: in vivo functional expression and biochemical characterization of glucans hydrolyzates analysis

**DOI:** 10.1186/s12896-015-0215-z

**Published:** 2015-10-19

**Authors:** Wei Wei, Jing Ma, Si-Qi Chen, Xiang-Hai Cai, Dong-Zhi Wei

**Affiliations:** Newworld Institute of Biotechnology, State Key Laboratory of Bioreactor Engineering, East China University of Science and Technology, 130 Meilong Road, 200237 Shanghai, People’s Republic of China

**Keywords:** Pullulanase type I, *Paenibacillus polymyxa*, Cold-adapted pullulanase, Functional expression, Enzymatic properties, Starch hydrolysis products

## Abstract

**Background:**

Pullulanase is an important debranching enzyme and has been widely utilized to hydrolyse the α-1,6 glucosidic linkages in starch/sugar industry. Selecting new bacterial strains or improving bacterial strains is a prerequisite and effective solution in industrial applications. Although many pullulanase genes have been cloned and sequenced, there is no report of *P. polymyxa* type I pullulanase gene or the recombinant strain. Meanwhile most of the type I pullulanase investigated exhibit thermophilic or mesophilic properties. There are just few reports of cold-adapted pullulanases, which have optimum activity at moderate temperature and exhibit rather high catalytic activity at cold. Previously, six strains showing distinct pullulan degradation ability were isolated using enrichment procedures. As containing novel bacterium resource and significant pullulanase activity, strain Nws-pp2 was selected for in-depth study.

**Methods:**

In this study, a type I pullulanase gene (pulN) was obtained from the strain P. polymyxa Nws-pp2 by degenerate primers. Through optimization of induced conditions, the recombinant PulN achieved functional soluble expression by low temperature induction. The enzyme characterizations including the enzyme activity/stability, optimum temperature, optimum pH and substrate specificity were also described through protein purification.

**Results:**

The pullulanase gene (named *pul*N), encoding a novel cold-adapted type I pullulanase (named Pul_N_), was obtained from isolated strain *Paenibacillus polymyxa* Nws-pp2. The gene had an open reading frame of 2532-bp and was functionally expressed in *Escherichia coli* through optimization of induced conditions. The level of functional Pul_N_-like protein reached the maximum after induction for 16 h at 20 °C and reached about 0.34 mg/ml (about 20 % of total protein) with an activity of 6.49 U/ml. The purified recombinant enzyme with an apparent molecular mass of about 96 kDa was able to attack specifically the α-1,6 linkages in pullulan to generate maltotriose as the major product. The purified Pul_N_ showed optimal activity at pH 6.0 and 35 °C, and retained more than 40 % of the maximum activity at 10 °C (showing cold-adapted). The pullulanase activity was significantly enhanced by Co^2+^ and Mn^2+^, meanwhile Cu^2+^ and SDS inhibited pullulanase activity completely. The *Km* and *Vmax* values of purified Pul_N_ were 15.25 mg/ml and 20.1 U/mg, respectively. The Pul_N_ hydrolyzed pullulan, amylopectin, starch, and glycogen, but not amylose. Substrate specificity and products analysis proved that the purified pullulanase from *Paenibacillus polymyxa* Nws-pp2 belong to a type I pullulanase.

**Conclusions:**

This report of the novel type I pullulanase in *Paenibacillus polymyxa* would contribute to pullulanase research from *Paenibacillus* spp. significantly. Also, the cold-adapted pullulanase produced in recombinant strain shows the potential application.

**Electronic supplementary material:**

The online version of this article (doi:10.1186/s12896-015-0215-z) contains supplementary material, which is available to authorized users.

## Background

The α-amylase family enzymes such as endo-amylases, glucoamylases, α-glucosidases and α-1,6-cleaving enzymes (pullulanase and isoamylase) have been widely used in starch-processing industries [1, 2]. Among these, pullulanase (EC 3.2.1.41), which can hydrolyze α-(1,6)-glucosidic linkages of pullulan or branched substrates is widely used in saccharification process for glucose, maltose, maltotriose and fructose production (usually used in combination with other amylolytic enzymes) [3–5]. Also, pullulanase was used in the fermentation industry to produce low carbohydrate “light beer” by adding pullulanase with fungal *α*-amylase or glucoamylase to the wheat during fermentation and bioethanol production [6]. Based on the substrate specificity and end product, pullulanase have been classified into five groups including (i) pullulanase type I (EC 3.2.1.41), also called the true pullulanases, specifically hydrolyses α-1,6 glycosidic linkages in pullulan or branched oligosaccharides to produce maltotriose and linear oligosaccharides (Additional file 1: Figure S1); (ii) pullulanase type II (amylopullulanase), attacks α-1,6 linkages in pullulan to produce maltotriose and α-1,4 linkages of other branched substrates to produce a mixture of glucose, maltose, and maltotriose (Additional file 1: Figure S1); (iii) pullulan hydrolase type I (neopullulanase, EC 3.2.1.135), hydrolyzing α-1,4 linkages in pullulan to produce panose (Glucose-α-1,6-Glucose-α-1,4-Glucose); (iv) pullulan hydrolase type II (isopullulanase, EC 3.2.1.57), hydrolyzing α-1,4 linkages in pullulan to produce isopanose (Glucose-α-1,4-Glucose- α-1,6-Glucose); and (v) pullulan hydrolase type III, attacks α-1,4 as well as α-1,6 glycosidic linkages in pullulan forming a mixture of maltotriose, panose and maltose [6–8].

Pullulanase is widely distributed in bacteria (*Bacillus* sp., *Aerobacter* sp., *Klebsiella* sp.), yeasts, fungi, plants and animals and most pullulanase are type II pullulanases [9]. A few type I pullulanases were investigated in gene level, such as *Bacillus flavocaldarius* KP 1228 [10], *Bacillus thermoleovorans* US105 [11], *Anaerobranca gottschalkii* [12], *Caldicellulosiruptor saccharolyticus* [13], *Bacillus* sp. CICIM 263 [14], *Thermotoga neapolitana* [15] and *Fervidobacterium pennavorans* Ven5 [16]. Most of type I pullulanase investigated exhibit thermophilic or mesophilic properties. There are just few reports of cold-adapted pullulanases, which have optimum activity at moderate temperatures and exhibit rather high catalytic activity at cold [8]. Cold-adapted enzymes are promising candidates for versatile biotechnological applications especially food industry due to the reduced risk of microbial contamination, minimized energy consumption and the fact that reacting compounds are often instable at increasing temperatures [17]. Among the most industrially relevant biocatalysts are starch-hydrolyzing enzymes, such as amylase, pullulanase, glucoamylase or α-glucosidase, that are widely used in food, feed, textile, pharmaceutical and detergent industries. Cloning the novel enzymes with distinct features, especially from easily grown bacterium, are of interest for industrial applications. *Paenibacillus polymyxa* strains (formerly *Bacillus polymyxa*) are well known for their ability to produce and secrete a large number of useful extracellular enzymes [18, 19]. Although many pullulanase genes from *Bacillus* spp. have been cloned and sequenced, there is no gene report of type I pullulanase in *P. polymyxa*. Castro et al. described the microbial characterization and enzymatic property in wild strain *Bacillus polymyxa* MIR-23 [20]. Kim et al. report the neopullulanase gene in *Paenibacillus* sp. KCTC 8848P which contained 510 amino acids [21].

Previously, six strains showing distinct pullulan degradation ability were isolated using enrichment procedures. As containing novel bacterium resource and significant pullulanase activity, strain Nws-pp2 was selected in-depth study. In this study, a type I pullulanase gene (*pul*N) was obtained from the strain *P. polymyxa* Nws-pp2 which was isolated from soil of fruit market garbage dump in Shanghai, China. Through optimization of induced conditions, the recombinant Pul_N_ achieved functional soluble expression by low temperature induction. The enzyme characterizations including the enzyme activity/stability, optimum temperature, optimum pH and substrate specificity were also described through protein purification. Another impressive fact is that the cold-adapted pullulanase showing low temperature catalytic performance (similar with enzymes from psychrophilic bacteria) was cloned from the mesophilic strain which grown well at ambient temperature (37 °C). Also, this report of the novel type I pullulanase from *P. polymyxa* with the detailed enzymatic properties would contribute to cold-adapted pullulanase research. The novel pullulanase showing cold-adapted biochemical characteristics provides the potential value in food-processing industry applications.

## Methods

### Bacterial strains/plasmids and chemicals

The bacterial strains, plasmids, chemicals, and culture conditions used in this study are listed in Additional file 1: Table S1 in supplemental material. Strain Nws-pp2 (named *Paenibacillus polymyxa* Nws-pp2, CCTCC AB 2013352) was isolated from soil of fruit market garbage dump in Shanghai, China. *Escherichia coli* DH5α (Invitrogen) and plasmid pMD19-T (TaKaRa) were used for gene cloning and sequencing. Plasmids pET-28a, pET-32a and pET-42a (Novagen) were the vectors used to construct the protein expression plasmid in *E. coli* BL21(DE3).

### Strain screening, *pul*N Cloning and gene analysis

Using pullulanase screening culture, the microbes showing obviously hydrolysis circle (treated with alcohol) were isolated, purified and transferred to maintenance slants. Among these, strain Nws-pp2 showing high ratios of hydrolysis circle were selected and identified by 16S rDNA gene analysis [22, 23].

**Fig. 1:**
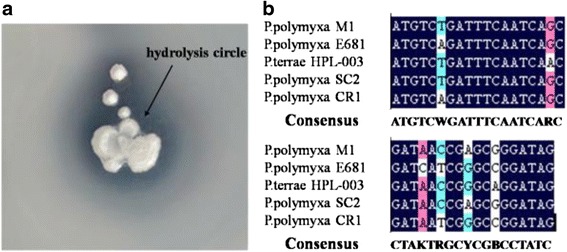
Pullulanase in wild strain and degenerate primers design. **a** Hydrolysis circle on pullulanase screening culture. **b** Comparison of five pullulanase genes from different resource. Sequences listed include partial of type I pullulanase genes from *Paenibacillus polymyxa* M1 (HE577054.1), *Paenibacillus polymyxa* E681 (CP000154.1), *Paenibacillus terrae* HPL-003 (CP003107.1), *Paenibacillus polymyxa* SC2 (CP002213.1) and *Paenibacillus polymyxa* CR1 (CP006941.1)

Based on the information of *Paenibacillus* spp. pullulanases in GeneBank, degenerate primers (pul-U:ATGTCWGATTTCAATCARC, pul-D:CTATCCBGCYCGRTKATC) for the pullulanase gene sequence were designed. According to NCBI search, the following gene sequences of predicted pullulanase from *Paenibacillus* spp. were analyzed to design degenerate primers (Fig. 1b).

The pullulanase gene (Genbank accession number: KJ740392) was amplified by PCR using primers pul-U/pul-D. The purified PCR product (2.5 kb) was cloned into pMD19-T and sequenced in two directions. The nucleotide sequence and predicted amino acid sequence were analyzed by the programs of Blast (NCBI, http://blast.ncbi.nlm.nih.gov/Blast.cgi). Enzyme Mw and pI were predicted using the ExPASy proteomic server program (http://web.expasy.org/compute_pi/). Multiple sequence alignments were performed using ClustalX/DNAMAN and the three-dimensional structure of this enzyme was predicted by the SWISS-MODEL server (http://swissmodel.expasy.org/SWISS-MODEL.html) [22].

According to the gene analysis, we designed other primers for pET family vectors. For *E. coli* expression, the gene was amplified by the primers (pul-pET-U:CGCGGATCCATGTCTGATTTCAATC, pul-pET-D:CCCAAGCTTCTATCCCGCTCGATCA). After digestion by *Bam*HI/*Hind*III restriction enzyme, the *pul*N gene was reclaimed and connected with pET-28a, pET-32a and pET-42a vectors respectively, which were digested by the same restriction endonuclease. The positive clones were selected by restriction enzyme digestion and finally confirmed by sequencing (Additional file 1: Figure S2). The recombinant plasmids pET-28-*pul*N, pET-32-*pul*N and pET-42-*pul*N were transformed into *E. coli* BL21(DE3). Recombinant cells (BL21-pET-28-*pul*N, BL21-pET-32-*pul*N and BL21-pET-42-*pul*N) were grown to saturation in LB medium supplemented with appropriate antibiotic.

### Induced conditions optimization and recombinant protein purification

When the optical density at OD_600_ nm reached 0.6, IPTG was added to induce the *lac* promoter of recombinant strains. Recombinant cells in different pET plasmid (BL21-pET-28-*pul*N, BL21-pET-32-*pul*N and BL21-pET-42-*pul*N) were firstly selected by SDS-PAGE and enzyme analysis (induction temperature: 37 °C, induction time 24 h, IPTG concentration: 0.5 mM). The conditions of *pul*N overexpression (BL21-pET-28-*pul*N) were optimized testing different parameters: temperatures (20, 25, 30 and 37 °C), the IPTG concentrations (0.1–1.5 mM, 0.1 mM gradient increasing) and induction time (2, 4, 8, 16, 24, and 32 h).

To show the expression level of recombinant pullulanase in each sample, we diluted the precipitated cells samples in 100 mM sodium phosphate buffer (pH 7.0) to a final concentration OD_600_ value equal to 10. All samples including supernatant of cell lysate and precipitation of cell lysate were dispersed in sodium dodecyl sulfate-polyacrylamide gel electrophoresis (SDS-PAGE) sample buffer and heated to 100 °C. Finally, proteins were separated by SDS-PAGE (12.5 % acrylamide/bis-acrylamide) and stained with coomassie brilliant blue. Moreover, the uninduced transformant and transformant harboring the empty plasmid were used as negative control. Purification steps of the pullulanase were performed at 4 °C [22].

### Enzyme properties analysis

The activity of Pul_N_ in pullulan hydrolysis were measured in triplicate at 35 °C in 100 mM citrate buffer (pH 6.0) using 3,5-dinitrosalicylic acid method. Enzyme activity was determined by measuring the enzymatic release of reducing sugar from pullulan. In this assay, 250 μl of 1 % (wt/vol) pullulan (sigma) solution and 175 μl of 100 mM citrate buffer were mixed and preincubated at 35 °C in a water bath. Then 75 μl of appropriately diluted enzyme sample was added into the preheated solution and the 500 μl reaction mixture was further incubated at 35 °C for 20 min. The reaction was stopped by addition of 750 μl 3,5-dinitrosalicylic acid reagent (DNS), followed by boiling for 15 min and cooled with ice-water mixture. The reaction system with the same enzyme sample added after DNS reagent was treated as the control. Activities were expressed as mean values in U (mg protein). 1 U is defined as the amount of enzyme that released 1 μmol reducing sugars (equivalent to glucose) per min under the assay conditions specified. All the pullulanase activities were converted to the whole fermentation volume (volumetric activities U or mg per ml medium). Each experiment was repeated three times and each experiment included three replicates. The average values of triplicate measurements were used as each activity value. The statistical tool Origin 8.0 was used to analyze the enzymatic properties data. All values are means ± SD from three independent experiments (repeats with SD of ≤ 5 %).

The pH optimum for Pul_N_ enzyme activity was studied over a range from pH 4.0–10.0 with pullulan as the substrate (35 °C). The pH stability of the enzyme was determined by incubating the enzyme in different buffers for 90 min and incubated at 35 °C. The following buffer systems were used: 100 mM citrate buffer (pH 4.0–6.0), 100 mM sodium phosphate buffer (pH 6.0–7.5), 50 mM Tris-HCL buffer (pH 7.5–8.5) and 100 mM glycine-NaOH buffer (pH 8.5–10.0). The temperature optimum for the Pul_N_ enzyme activity was assayed at 10–75 °C (pH 6.0). The thermal stability of Pul_N_ was evaluated by assaying its residual activity after incubation of the enzyme at various temperatures (35, 40, 45 and 50 °C) for 300 min in sodium phosphate buffer (pH 6.0). The enzyme sample without any incubation was considered as control (100 %).

Effects of metal ions (1, 5 and 10 mM) and other reagents on enzyme activity were determined in a standard assay medium. The reaction mixtures containing the enzyme sample were incubated at 35 °C for 60 min in 100 mM citrate buffer, pH 6.0. The enzyme sample without any additives was considered as control (100 %).

### Kinetic parameters measurements

The kinetic parameters *Km* and *Vmax* were determined for the substrate pullulan at 35 °C in 0.1 M citrate buffer (pH 6.0) using DNS method. Different concentrations of pullulan (2, 4, 6, 8, 10 and 12 mg/ml) were used in the standard reaction system. Samples were withdrawn from reaction system (2 min interval) and measured. The initial velocity values (the amount of maltotriose generated per minute) and substrate concentrations were transformed into Lineweaver-Burk reciprocal plots. Then, *Km* and *Vmax* were calculated from the slopes of the curves (Additional file 1: Figure S4).

### Characterization of substrates specificity and hydrolysis products

The ability of the purified enzyme to hydrolyze various carbohydrates was examined at 35 °C and pH 6.0 in 100 mM citrate buffer. The carbohydrates tested were pullulan, soluble starch, amylose, amylopectin and glycogen at a concentration of 1 % (w/v). About 0.5 U enzyme was added to the different substrate, and the reaction mixture was incubated at 35 °C for 8 h. Subsequently, the reaction systems were stopped by boiling for 5 min and centrifugation for later use. The hydrolysis products on different substrate were analyzed by thin-layer chromatography (TLC) and high-performance liquid chromatography (HPLC), respectively. Silica gels plate was used for TLC analysis. After activated at 110 °C for 1 h, prepared samples were spotted onto the plate. The plate was placed in a chamber containing a solvent system of acetic acid-N-butanol-water (1:2:1) for a while and then was withdrawn. After the plate was dried, the reducing sugar was detected using sulfuric acid solution (containing 0.3 % N-1-naphthyl-ethylenediamine and 5 % H_2_SO_4_ in methanol) at 110 °C for 10 min. The cold-adapted enzymatic property of Pul_N_ was confirmed by TLC with the industrial enzyme as a control (Promozyme; Novozymes A/S). The enzymes (about 0.5 U) were added to the pullulan substrate, and the reaction mixture was incubated at 0, 20 and 40 °C for 120 min. The hydrolysis products were analyzed by thin-layer chromatography (TLC). Waters analysis column (Ultmate XB-NH2-3.5 μm, USA) with a refractive index detector were used for HPLC. Solvent system of double-distilled water-acetonitrile (20:80) was used as the mobile phase at a flow rate of 0.8 ml/min. The temperature of column and refractive index detector were 45 and 40 °C, respectively. The sample quantity was 10 μl each time.

In order to distinguish maltotriose (only α-1,4 bonds) from panose or isopanose (α-1,4 and α-1,6 bonds), the incubation was performed with α-glucosidase in a 100 mM sodium phosphate buffer (pH 6.0) at 37 °C. This enzyme is capable of hydrolyzing α-1,4 but not α-1,6 linkages in short-chain oligosaccharides.

### Nucleotide sequence accession numbers

The GeneBank accession numbers for the *Paenibacillus polymyxa* Nws-pp2 *pul*N gene is KJ740392.

## Results

### Isolation and identification of bacterium

Strain Nws-pp2 was isolated from soil sample of fruit market garbage dump and showed the pullulanase activity when cultivated on the pullulanase screening culture medium. After 48 h growing on the pullulanase screening culture medium plate at 30 °C, significant hydrolysis circle appeared around the bacterial colony (treated with alcohol) (Fig. 1a). The strain was identified as *P. polymyxa* according to the result of 16S rDNA phylogenetic analysis and the morphology. The 16 s rDNA (1443-bp) displayed the similarity of 99 % to *P. polymyxa* 1851 (Accession no. EU982546) and *P. polymyxa* M1 (Accession no. EF656457). The strain was renamed as *P. polymyxa* Nws-pp2 and deposited at the China Center for Type Culture Collection (http://www.cctcc.org/, Wuhan, China. Accession number: CCTCC AB 2013352).

### Cloning of the *pul*N and gene analysis

Through PCR amplification, a 2532-bp DNA fragment encoding a polypeptide of 843 amino acids was cloned and sequenced. The GenBank accession number of *pul*N is KJ740392. The G + C content (%) of the *pul*N is 53.6 %. Homology analysis revealed that Pul_N_ in *P. polymyxa* Nws-pp2 is 88 % identical to the CP002213.1 in *P. polymyxa CR1* (hypothetical type I pullulanase), 87 % identical to the CP000154.1 in *P. polymyxa* E681 and 82 % identical to the CP003107.1 in *P. terrae* HPL-003. The molecular weight of Pul_N_ was estimated to be 95.6 kDa, and the pI value was calculated to be 4.92 by the ExPASy compute pI/Mw program algorithm. Meanwhile, as no report of this pullulanase in previous research, the three-dimensional structure of Pul_N_ was predicted by the SWISS-MODEL server and the protein structure was viewed by PdbViewer (Additional file 1: Figure S3).

The sequence alignment of the conserved region (Pul_N_) and pullulanase Type I from other different bacterial sources was performed and revealed conservation of aa in regions associated with catalysis and stabilization of the protein, e.g. the active site (Tyr^233^, Asp^234^, His^281^, Arg^345^, Asp^347^, Leu^348^, Glu^377^, Trp^379^, Asp^405^, Arg^408^, His^458^, Asp^459^, Asn^460^, Asn^551^, Tyr^553^), the catalytic site (Asp^347^, Glu^377^, and Asp^459^) and the putative carbohydrate-binding site (Trp^759^, Trp^761^, Ile^796^, Lys^805^, Asp^810^).

**Fig. 2:**
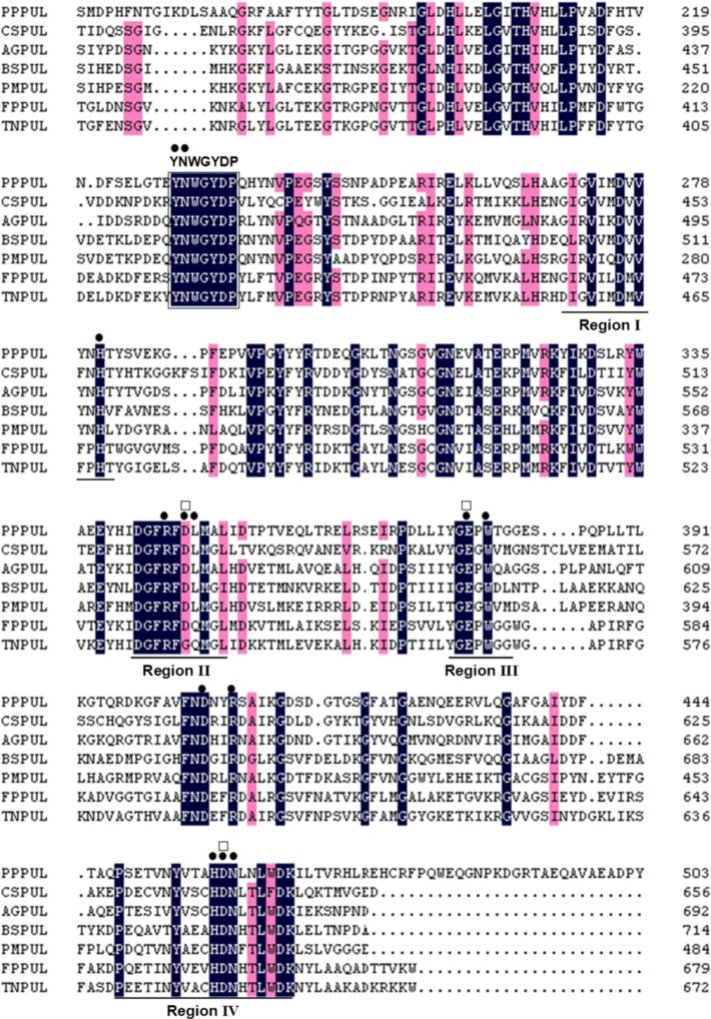
Sequence alignment of the conserved region of Pul_N_ and pullulanase Type I from other different bacterial sources. The sequences used in this alignment were obtained from GenBank as following. PPPUL: *P. polymyxa* pullulanase (this study; GenBank accession no. KJ740392); CSPUL: *C. saccharolyticus* pullulanase (GenBank accession no. L39876); AGPUL: *A. gottschalkii* pullulanase (GenBank accession no. AY541591); BSPUL: *Bacillus* sp. CICIM 263 pullulanase (GenBank accession no. AGA03915); PMPUL: *P. mucilaginosus* KNP414 pullulanase (GenBank accession no. YP004645603); FPPUL: *F. pennavorans* Ven5 pullulanase (GenBank accession no. AF096862); TNFUL: *T. neapolitana* strain KCCM 41025 pullulanase (GenBank accession no. FJ716701). Regions I, II, III, and IV are lined. YNWGYDP conserved in all type I pullulanases is boxed. The numbering refers to the amino acid position in each sequence. The structures are denoted as follows: ●, the active site; □, the catalytic site

Compared with the other detail reported type I pullulanase (Table 1), Pul_N_ from *P. polymyxa* Nws-pp2 shows low sequence homology. Pul_N_ has the highest amino acid homology (47.6 %) with rPulAg from the *Anaerobranca gottschalkii*, and 35.5 % homology to the type I pullulanase from *Paenibacillus mucilaginosus.* Although the overall similarity value is very low, a highly conserved region consisting of seven amino acids (YNWGYDP) is found in all type I pullulanases [6, 16, 22, 23]. This highly conserved seven-residue was detected at the position from 229 to 235 on the N-terminal side of Pul_N_ in this study (Fig. 2). Type II pullulanase (with α-1,6 and α-1,4 glycoside activities) gene sequences do not contain this conserved region. This result revealed that Pul_N_ belong to a type I pullulanase. Meanwhile, four conserved regions (region I, II, III, IV) that are common to the glycoside hydrolase family enzymes(GH13) were also identified in Pul_N_ (Fig. 2).

**Table 1:** Pairwise similarity between the type I pullulanase amino acid sequences

Similarity value (%) for pullulanase sequence from:							
Pullulanase sequence from:	*P.polymyxa* Nws-pp2	*C.saccharolyticus*	*A.gottschalkii*	*Bacillus* sp. CICIM 263	*P.mucilaginosus*	*F.pennavorans* Ven5	*T.neapolitana*
*Paenibacillus polymyxa* Nws-pp2	100	37.3	47.6	38.5	41.6	39.1	38.9
*Caldicellulosiruptor saccharolyticus*		100	43.2	36.8	40.0	37.3	41.1
*Anaerobranca gottschalkii*			100	41.3	44.4	43.8	44.4
*Bacillus* sp. CICIM 263				100	52.7	39.8	39.6
*Paenibacillus mucilaginosus*					100	41.5	42.6
*Fervidobacterium pennavorans* Ven5						100	68.0
*Thermotoga neapolitana*							100

The sequences are from the following sources: *P. polymyxa* Nws-pp2 (this study; GenBank accession no. KJ740392); *C. saccharolyticus* (GenBank accession no. L39876); *A. gottschalkii* (GenBank accession no. AY541591); *Bacillus* sp. CICIM 263 (GenBank accession no. AGA03915); *P. mucilaginosus* (GenBank accession no. YP004645603); *F. pennavorans* Ven5 (GenBank accession no. AF096862); *T. neapolitana* (GenBank accession no. FJ716701)

### Gene expression in *E. coli* and optimization of the culture conditions

**Fig. 3:**
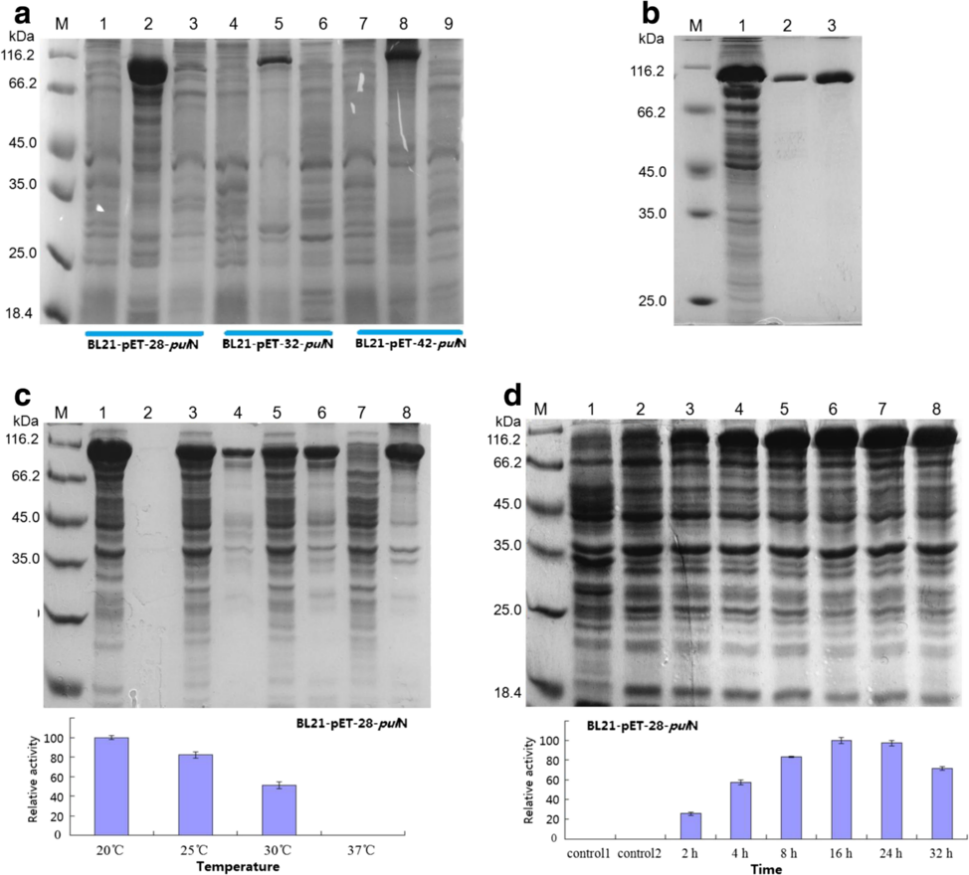
SDS-PAGE of the recombinant protein in *E. coli.* **a** SDS-PAGE of recombinant Pul_N_ in different pET plasmid (induction temperature: 37 °C, induction time 24 h, IPTG concentration: 0.5 mM)*.* Lane M: protein molecular weight markers; Lane 1: supernatant without inducer (BL21-pET-28-*pul*N); lane 2-3: precipitation and supernatant; lane 4: supernatant without inducer (BL21-pET-32-*pul*N); lane 5-6: precipitation and supernatant; lane 7: supernatant without inducer (BL21-pET-42-*pul*N); lane 8-9: precipitation and supernatant. **b** SDS-PAGE of the purified Pul_N_. **c** Recombinant Pul_N_ production in BL21-pET-28-*pul*N with different induction temperatures (IPTG concentration: 0.1 mM, induction time 24 h). lane 1-2: supernatant and precipitation at 20 °C; lane 3-4: supernatant and precipitation at at 25 °C; lane 5-6: supernatant and precipitation at 30 °C; lane 7-8: supernatant and precipitation at at 37 °C. The histogram shows the comparison of pullulanase activity at different induction temperature. **d** Recombinant Pul_N_ production in BL21-pET-28-*pul*N with different induction time (IPTG concentration: 0.1 mM, induction temperature 20 °C). lane 1: supernatant of empty vector, lane 2: supernatant of BL21-pET-28-*pul*N without inducer; lane 3-8: supernatant of recombination strain after 2 h, 4 h, 8 h, 16 h, 24 h, 32 h induction (BL21-pET-28-*pul*N). The histogram shows the comparison of pullulanase activity after different induction time

The *E. coli* expression vectors pET-28a, pET-32a and pET-42a were used to express foreign proteins in this study. The SDS-PAGE profile revealed the presence of an additional protein band in all recombinant strains with an estimated molecular mass of 99 kDa (BL21-pET-28-*pul*N), 113 kDa (BL21-pET-32-*pul*N, with 109 aa Trx•Tag protein) and 128 kDa (BL21-pET-42-*pul*N, with 220 aa GST•Tag protein), which was closed to the molecular mass of Pul_N_. The SDS-PAGE results showed that the recombinant protein appeared mostly as inclusion bodies (induction temperature: 37 °C, induction time 24 h) in recombinant strains BL21-pET-28-*pul*N/BL21-pET-32-*pul*N/BL21-pET-42-*pul*N and no pullulanase activity was detected. Using low temperature induction, the recombinant protein appeared partly as a soluble protein (Fig. 3a). As showing the highest expression level and pullulanase activity, recombinant strain BL21-pET-28-*pul*N was used to detect the enzymatic properties in the next study.

The optimization of Pul_N_ (BL21-pET-28-*pul*N) expression conditions was concerned using the single-factor experiments method. As shown in Fig. 3c, high induction temperature had a negative effect on the production of soluble recombinant protein. When the induction temperatures was 25 °C or above (Fig. 3c, line 3-8), the content of soluble protein existed in supernatant declined gradually (Fig. 3c, line 3, 5 and 7). Meanwhile, inclusion bodies (lack of proper folding) existed in precipitate increased (Fig. 3c, line 4, 6 and 8). Meanwhile, enzyme activity decreased when induction temperature increased, and no pullulanase activity can be detected when the temperature up to 37 °C, especially. These results suggested that low culture temperature facilitated proper folding of the protein and 20 °C was regarded as the optimal temperature for the production of the recombinant Pul_N_. Fermentation time was another remarkable parameter that affected the production of Pul_N_. As shown in Fig. 3d, the expressed protein Pul_N_ increased with the extension of the culture time within a certain range (Fig. 3d, line 3-6). Also, the pullulanase activity was increased with the recombinant protein expression level. However, when cells were fermented for more than 16 h, there was no more significant accumulating of the recombinant protein (Fig. 3d, line 7 and 8). So we chose 16 h as the culture time to collect recombinant strains (BL21-pET-28-*pul*N). The *E. coli* BL21(DE3) harboring pET28a empty plasmids and recombinant strains without IPTG induction were fermented in same condition as controls (Fig. 3d, line 1 and 2). No pullulanase activity was detected in uninduced transformant or from transformant harboring the empty pET plasmid. The influence of IPTG concentration (from 0.1 to 1.0 mM) and the shaking speed (150, 180, 200 and 230 rpm) on the target protein distribution were negligible. However, pullulanase activity decreased remarkable when IPTG concentration was above 1.0 mM (data not shown). The optimal expression conditions were detected with 0.1 mM IPTG, 20 °C for induction temperature and induction time for 16 h in our research. The recombinant pullulanase (Pul_N_) reached about 0.34 mg/ml (about 20 % of total protein). The optimal enzyme activities of recombinant Pul_N_ was 6.49 U/ml.

### Purification and quantitative assay of recombinant Pul_N_ by SDS-PAGE analysis

The recombinant pullulanase (Pul_N_ from strain BL21-pET-28-*pul*N) was overexpressed in *E. coli* BL21(DE3) cells under the optimal conditions and was purified by Ni-NTA purification procedures (Fig. 3). The BL21-pET-28-*pul*N construct contained His-tag in order to facilitate Ni-NTA purification. About 10.6 mg Pul_N_ which was purified up to 4.28 times with a recovery of 58.7 % was obtained using Ni-NTA affinity chromatography (Table 2). The crude extract enzyme specific activity was 3.78 U/mg, and specific activity was up to 16.17 U/mg after purification. The purified enzyme migrated on SDS-PAGE as a single band with an apparent molecular mass of about 99.0 kDa (Fig. 3b). There was no obvious bands of impurity protein appeared after being concentrated five times (Fig. 3b). The results indicated that Ni-NTA affinity chromatography was an appropriate method for the protein purification (Table 2).

**Table 2:** Purification of Pul_N_ by Ni-NTA affinity chromatography

Purification step	Total activity (U)	Total protein (mg)	Specific activity (U/mg)	Yield (%)	Purification fold
Crude extract	292.05	77.26	3.78	100.00	1.00
Ni-NTA	171.41	10.60	16.17	58.7	4.28

The crude enzyme was obtained from 45 ml culture and 10 ml purified enzyme was obtained.

### Biochemical characterization of the purified recombinant pullulanase

**Fig. 4:**
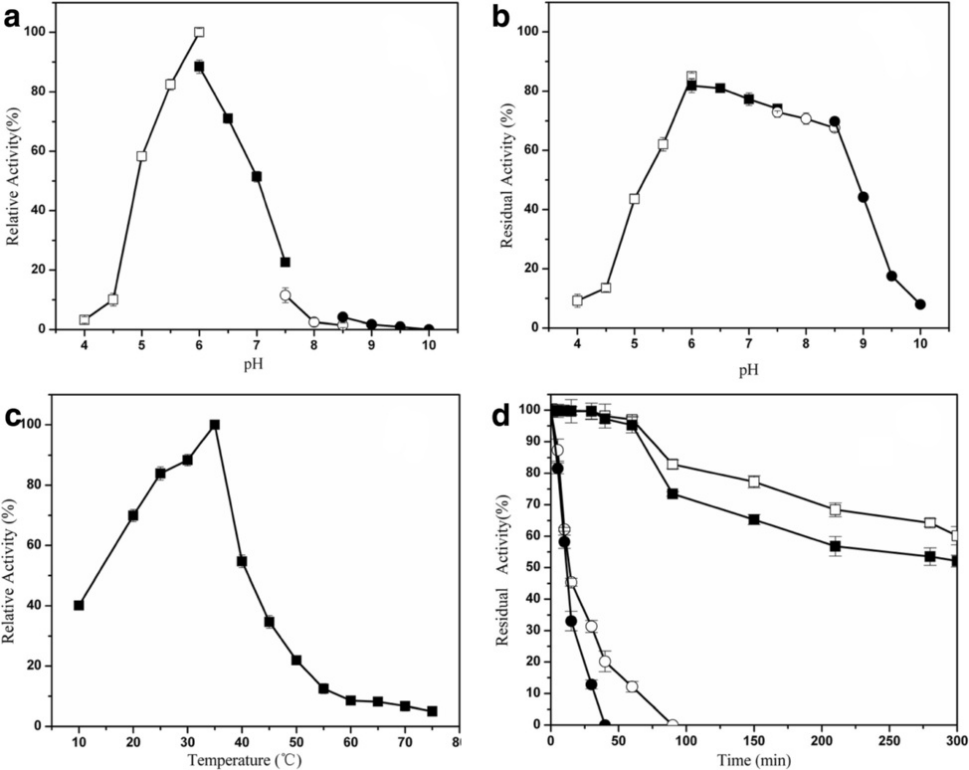
Characterization of recombinant Pul_N_. **a** Effect of pH on Pul_N_ activity at 35 °C in buffers ranging from pH 4.0-10.0. **b** Effect of pH on the stability of Pul_N_ activity. After incubation at 35 °C for 90 min in buffers ranging from pH 4.0-10.0 and the residual activity was measured at pH 6.0. **c** Effect of temperature on Pul_N_ activity assayed at pH 6.0. **d** Thermostability of Pul_N_. The enzyme was preincubated at 35 °C (□), 40 °C (■), 45 °C (○), 50 °C (●) at pH 6.0. After various time intervals, samples were withdrawn and the residual activity was measured at 35 °C. The statistical tool Origin 8.0 was used to analyze the enzymatic properties data. Each value represents the mean ± SD of triplicate

The activities of pullulanase at various pH values and temperatures were measured by using pullulan as the substrate. The purified Pul_N_ exhibited a higher enzyme activities over a pH range of 5.0–6.5, and the highest specific enzyme activity was detected at pH 6.0 (Fig. 4a). The enzyme retained more than 62 % of the maximal activity after incubation in pH 5.5–8.5 for 90 min (Fig. 4b). The optimal temperature of purified Pul_N_ was 35 °C, and there was still 40 % of the maximal activity at only 10 °C (Fig. 4c). Pul_N_ retained more than 61 % of the initial activity after incubation at 35 °C for 300 min, and more than 52 % of the initial activity after incubation at 40 °C for 300 min, exhibiting a remarkable stability. However, the activity decreased sharply when the temperature is over 45 °C, it was completely inactivated at 45 °C for 90 min and at 50 °C for 40 min, respectively (Fig. 4d).

The effects of different metal ions and chemical reagents on the enzyme were examined in pH 6.0 citrate buffer at 35 °C for 15 min (Tables 3 and 4). At low concentration (1 mM), Ca^2+^, Zn^2+^, Ni^2+^ partly inhibited or had no effects on the enzyme, while Mn^2+^, Co^2+^could slightly enhanced the enzyme activity. However, at high concentration (5 and 10 mM), Zn^2+^ and Fe^2+^ showed significantly inhibited effects, reducing the activity to 83.8, 49.3, 58.3 and 42.0 % of the control samples, respectively. In comparison, Mg^2+^ could slightly enhance the enzyme activity. Cu^2+^ showed significantly inhibited effects and only 2.9 % pullulanase activity was detected within 10 mM Cu^2+^. The pullulanase activities are not significantly affected by 5 mM EDTA, indicating that metal ions were not required for enzyme activity. The enzyme was unstable during incubation at 35 °C for 15 min in the presence of surfactants such as 2 and 4 M urea and 1 % SDS, which led to significant loss of activity. Enzyme activity was enhanced by the addition of reducing agents such as dithiothreitol (DTT) and 2-mercaptoethanol, and was inhibited by α-cyclodextrins, which is known as possible competitive inhibitors of this enzyme [16].

**Table 3:** Effects of metal ions and chemical reagents on the Pul_N_ activity

Metal ions compounds	Relative activity (%)		
	1 mM	5 mM	10 mM
None	100.0	100.0	100.0
MgCl_2_	104.1 ± 1.4	109.0 ± 0.2	117.0 ± 0.5
CaCl_2_	97.9 ± 2.0	97.4 ± 1.6	96.0 ± 1.6
CuCl_2_	75.1 ± 2.5	11.9 ± 0.5	2.9 ± 0.1
ZnSO_4_	97.6 ± 0.7	83.8 ± 0.7	49.3 ± 1.4
NiSO_4_	99.0 ± 2.3	103.1 ± 0.2	102.1 ± 0.9
MnCl_2_	114.8 ± 2.0	154.8 ± 2.7	215.2 ± 4.8
CoCl_2_	121.7 ± 0.7	124.2 ± 1.1	125.1 ± 0.9
FeSO_4_	77.2 ± 3.4	58.3 ± 0.9	42.0 ± 1.1

The data represent the mean of three experimental repeats with SD of ≤ 5 %

**Table 4:** Effects of metal ions and chemical reagents on the Pul_N_ activity

Reagents	Concentration	Relative activity (%)
None	-	100
EDTA	5 mM	102.1 ± 0.2
SDS	1 mM	12.3 ± 0.8
TritonX-100	0.1 %	99.8 ± 1.1
	1 %	99.4 ± 0.2
Urea	2 M	71.2 ± 0.4
	4 M	34.8 ± 2.3
2-Mercaptoethanol	5 mM	132.7 ± 1.9
	10 mM	167.3 ± 4.0
DTT	10 mM	154.4 ± 1.5
α-cyclodextrin	0.1 %	77.8 ± 0.8

The data represent the mean of three experimental repeats with SD of ≤ 5 %

The kinetics (*Vmax* and *Km*) of the purified pullulanase was determined by Lineweaver-Burk double reciprocal plot at varying substrate (pullulan) concentration. The *Km* of the enzyme with pullulan as substrate was 15.25 mg/ml, and the *Vmax* was 20.1 U/mg.

### Substrate specificity and analysis of hydrolyzates

**Fig. 5:**
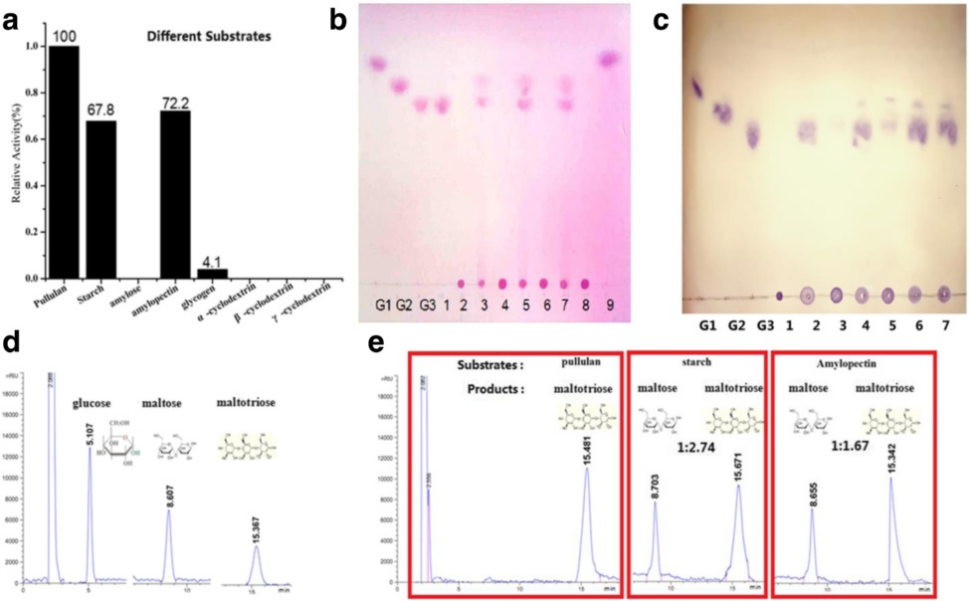
Substrate specificity and analysis of hydrolyzed products. **a** Relative activity on different substrates. **b** TLC analysis of hydrolysis products with various substrates. Lane G1: glucose; lane G2: maltose; lane G3: maltotriose; lane 1: pullulan with Pul_N_; lane 2: pullulan; lane 3: starch with Pul_N_; lane 4: starch; lane 5: amylopectin with Pul_N_; lane 6: amylopectin; lane 7: glycogen with Pul_N_; lane 8: glycogen; lane 9: products of pullulan with α-glucosidase. **c** TLC analysis of hydrolysis products compared with the industrial enzyme at different temperature. Lane G1: glucose; lane G2: maltose; lane G3: maltotriose; lane 1: pullulan; lane 2: pullulan with Pul_N_ at 0 °C; lane 3: pullulan with Promozyme at 0 °C; lane 4: pullulan with Pul_N_ at 20 °C; lane 5: pullulan with Promozyme at 20 °C; lane 6: pullulan with Pul_N_ at 40 °C; lane 7: pullulan with Promozyme at 40 °C. **d** HPLC analysis of standard (glucose, maltose, maltotriose). **e** HPLC analysis of hydrolysates from with various substrates (pullulan, soluble starch and amylopectin)

The ability of Pul_N_ to hydrolyze various α-glucans as well as pullulan was determined by using various substrates at concentrations of 1 % (w/v). The activity of hydrolyzing pullulan, amylopectin, starch and glycogen ]were 16.17 U/mg, 11.67 U/mg, 10.96 U/mg and 0.65 U/mg, respectively. No activity was detected with amylose and cyclodextrin as substrate. Pul_N_ can hydrolyze pullulan, soluble starch, amylopectin, glycogen, all of which have α-1,6 glycosidic linkages in their structures (Fig. 5a). In comparison, Pul_N_ cannot hydrolyze amylose, which only consist of α-1,4 glycosidic linkage.

The hydrolyzates of enzyme substrate reaction were detected by TLC and HPLC using standard sugar solutions (glucose, maltose, maltotriose) (Fig. 5b,d). After incubation of this enzyme with pullulan, the hydrolytic pattern revealed the complete conversion of pullulan to maltotriose in an endo-acting fashion. The hydrolyzed product of pullulan was subsequently incubated with bacteria α-glucosidase, which specifically hydrolyzes α-1,4 glycosidic linkages. A complete conversion into glucose verified that the hydrolyzed product from pullulan was maltotriose, not panose or isopanose (possessing α-1,4 and α-1,6 glycosidic linkages). The results indicated that pullulan (contain only α-1,6 linkages) was completely converted to maltotriose (Fig. 5b,d). As to soluble starch and amylopectin, the major hydrolysis products were maltotriose and maltose (The molar ratio of maltotriose: maltose was 2.74:1 and 1.67:1, respectively) (Fig. 5e). According to these results, Pul_N_ specifically attacks α-1,6 linkages of branched oligosaccharides and can therefore be classified as a type I pullulanase. Meanwhile, the results (pullulan hydrolytic reactions with the industrial enzyme as a control at different temperature) revealed that cold-adapted pullulanase (Pul_N_) shows the better property (hydrolytic activity) under low temperature (Fig. 5c).

## Discussion

Pullulanase, an important debranching enzyme, has been widely utilized to hydrolyse the α-1,6 glucosidic linkages in starch/sugar industry and used predominantly in conjunction with other enzymes that break down starch [1]. Selecting new bacterial strains or improving bacterial strains is a prerequisite and effective solution in industrial applications and will be important for maximal production in order to get better yield in starch industry [5, 6]. Although *P. polymyxa* strains are well known for their ability to secrete a large number of useful extracellular enzymes, there is no report of *P. polymyxa* type I pullulanase gene and the recombinant strain. Type I pullulanase is an enzyme that specifically hydrolyses α-1,6 glycosidic linkages in pullulan or branched oligosaccharides to produce maltotriose and linear oligosacharides [6]. Most of type I pullulanase have been investigated exhibit thermophilic or mesophilic properties. There are just few reports of cold-adapted pullulanases. In a previous study, a pullulanase, Pul-SH3, (about 70 kDa) purified from the optimized culture of a wild strain *Exiguobacterium* sp. SH3 showed high catalytic activity at ambient temperature and seemed to exhibit outstanding cold-adapted characteristics [8]. However, the gene of the enzyme was not obtained and the enzymatic properties were not characterized further. In this paper, the novel cold-adapted type I pullulanase from *P. polymyxa* was obtained and expressed in *E. coli*. The enzyme activity reached 6.49 U/ml after a series of induction optimization. Also, biochemical characterization and substrate specificity of the purified recombinant enzyme were detail described.

Amylolytic enzymes have different catalytic pro perties and structures. However, as containing similar substrates and modes of action, strong similarities exist in their amino acid sequences between substrate binding sites and catalytic sites. BLASTP analysis of the amino acid sequence derived from the *P. polymyxa* pullulanase (Pul_N_) revealed the YNWGYDP motif which is known to be involved in the hydrolysis of α-1,6 bonds in pullulan and the four conserved regions (regions I, II, III, and IV) that are typical of glycosyl hydrolase family (GHF) 13 α-amylases. These regions contribute to the active site architecture within the catalytic domain of GHF 13 enzymes and encompass the catalytic triad of acidic residues, namely Asp^347^, Glu^377^, and Asp^459^ in the *P. polymyxa* pullulanase (Additional file 1: Figure S3b). The pattern of pullulanase action against pullulanase substrates differs from that for α-amylases, indicating that other amino acids in addition to the three residues mentioned above are involved in catalysis. Tyr^233^ and Trp^231^, located in the pullulanase sequence YNWGYDP, are important for van der Waals interactions with glucose residues in subsites -1 and -2 [15]. Moreover, the histidine (His) residues, which have been found to participate specifically in catalysis of α-1,6 glycosidic linkages by *Klebsiella aerogenes* pullulanase [24], are also present in *P. polymyxa* pullulanase in regions I and IV at positions His^498^ and His^676^, respectively.

As showing the highest expression level and pullulanase activity, recombinant strain BL21-pET-28-*pul*N was used to express foreign proteins and detect the enzymatic properties in this study. In this work, some culture conditions affected enzyme activity significantly. The optimal expression conditions were detected with 0.1 mM IPTG, 20 °C for induction temperature and induction time for 16 h in our research. High temperature (25, 30, 37 °C) and/or high IPTG concentration (>1.0 mM) resulted in activity decreasing. In this study, pullulanase gene (*pul*N) was functionally expressed in *E. coli* through expression vector replacement and induction expression strategy optimization. Another impressive fact is that through the optimization of expression, most of the inclusion bodies (with no pullulanase activity, 37 °C) were transformed to soluble protein (functional expression, 20 °C) distinctly (Fig. 3).

This study provided the detailed Pul_N_ enzymatic properties and the worth concern was the low temperature catalytic performance (similar with enzymes from psychrophilic bacteria, but cloned from the mesophilic strain). As we known, most of type I pullulanase shows thermophilic or mesophilic properties (Table 5) and the enzyme activity of most thermophilic/mesophilic pullulanases decline dramatically in lower temperatures (below their optimum temperature). A more comprehensive knowledge on the structure and function of the cold-adapted amylopullulanase from *Exiguobacterium* sp. SH3 set the stage for development of customdesigned biocatalysts that are efficient at ambient temperatures [8]. Addition of a novel cold-adapted glycogen branching enzyme from *Rhizomucor miehei* to wheat bread increased its specific volume and had an antistaling effect in comparison with the control [25]. However, there was few report of cold-adapted pullulanases (wild strain) [26] and no report of cold-adapted pullulanase produced from recombinant strain with detailed enzymatic properties (Table 5). In this research, purified pullulanase had an optimal temperature at 35 °C which was similar to that from *Exiguobacterium* sp. SH3 (30 °C) [8] and much lower than that from *Bacillus acidopullulyticus* (60 °C) [26], *Anaerobranca gottschalkii* (70 °C) [12], *Thermotoga neapolitana* (80 °C) [15] and *Fervidobacterium pennavorans* Ven5 (80 °C) [16]. Both pullulanase from *Exiguobacterium* sp. SH3 and Pul_N_ in this study has a 40 % residual activity when temperature is only 10 °C, exhibiting a good low temperature catalytic performance. The purified Pul_N_ was sensitive to high temperature, and it was inactivated within 40 min in 50 °C and 10 min in 60 °C. The thermal stability of Pul_N_ is much poorer than those from thermophilic microorganisms. Purified Pul_N_ had an optimal pH of 6.0 in citrate buffer. Similarly, optimal pH 6.0 was also observed from *Fervidobacterium pennavorans* Ven5 [16], *Bacillus* sp. AN-7 [27] and *Bacillus stearothermophilus* [28]. Pullulanase from other strains showed different optimal pH. The optimal pH of pullulanase was pH 4.5 from *Bacillus acidopullulyticus* [26], pH 6.5 from *Bacillus* sp*.* CICIM 263 [14], and pH 8.0 from *Anaerobranca gottschalkii* [12]. The best pH condition for starch saccharification is 4.5 to 5.5, and that is why pullulanase from *Bacillus acidopullulyticus* (Promozyme; Novozymes A/S) was wildly used. The purified Pul_N_ was stable after incubated for 90 min in pH condition from 5.5 to 8.5. The results indicated that although the activity of Pul_N_ in alkaline environment is low (55–6 % of the maximum activity), it has a considerable stability in alkaline environment. Mn^2+^, Co^2+^ could enhance the enzyme activity, but Zn^2+^, Fe^2+^ and Cu^2+^ inhibited the enzyme activity, just like pullulanase from *Fervidobacterium pennavorans* Ven5 [16]. Ca^2+^ was not inhibitory and was agreement with the pullulanase primary structure of Pul_N_ lacks typical Ca^2+^ binding residues found in other members of glycoside hydrolase family 13 [12]. In our research, the recombinant Pul_N_ activities are not significantly affected by 5 mM EDTA, and this results may indicated that divalent cation was not necessary in recombinant Pul_N_. The enzymatic properties (unaffected by EDTA) was also approved by the pullulanase in *Anaerobranca gottschalkii* (EDTA was not inhibitory even at high concentrations 0.5 M) [12]. Reducing agents such as dithiothreitol (DTT) and 2-mercaptoethanol cause a significant activation of the cloned pullulanase (Pul_N_). It is likely that DTT inhibits the oligomerization of the enzyme (leading to its inactivation) by reducing the disulfide linkage between enzyme monomers.

**Table 5:** The catalytic activity comparison with different temperature

Enzyme	Strain and reference	optimum temperature (°C)	Relative activity at a range of temperature (%)						
			10	20	30	40	50	60	70
Pul_N_	*Paenibacillus polymyxa* Nws-pp2 (this study)	35	40	70	85	55	<20	—	—
Pul-SH3	*Exiguobacterium* sp. SH3 [8]	30	45	80	100	70	<40	—	—
rPulAs	*Fervidobacterium pennavorans* Ven5 [16]	80	—	—	<10	20	35	50	80
rPulAg	*Anaerobranca gottschalkii* [12]	70	—	—	—	<30	55	85	100
pulullanase	*Bacillus* sp. AN-7 [27]	90	—	—	—	<25	50	65	80
pulullanase	*Bacillus stearothermophilus* [28]	65	—	<10	25	45	70	95	10
PulA1	*Bacillus sp.* CICIM 263 [14]	70	—	—	<40	50	60	80	100

The pullulanase from *P. polymyxa* Nws-pp2 preferentially hydrolyzed pullulan (Fig. 5) and other carbohydrates containing α-1,6 glycosidic linkages, including amylopectin (72.2 % activity relative to the pullulan-cleaving activity) (Fig. 5), soluble starch (67.8 %), and glycogen (4 %), while amylose was not significantly hydrolyzed. The substrate and product profiles (analysis by TLC and HPLC) indicate that Pul_N_ specifically attacks α-1,6 linkages of branched oligosaccharides and therefore can be classified as a type I pullulanase. Therefore, the pullulanase from *P. polymyxa* Nws-pp2 is the novel cold-adapted type I pullulanase from *Paenibacillus* sp. that has been described.

## Conclusions

In this study, we obtained a novel pullulanase gene from the strain *P. polymyxa* Nws-pp2 and the gene was functional expressed in *E. coli* through expression vector replacement and induction expression strategy optimization. This is the novel report of the type I pullulanase in *Paenibacillus polymyxa* (including wild strain and recombinant strain) and heterologous expression with the detailed enzymatic properties. Also, the novel cold-adapted pullulanase provide the potential value in food industry applications. Future investigations will focus on the application of this enzyme in the food industry and studies on structure-function relationships.

## Additional file

Additional file 1:
**Section A:** The schematic representation of type I pullulanase.** Section B:** The bacterial strains, plasmids, chemicals and culture conditions. **Section C:** Pullulanase gene manipulation/propagation. **Section D:** The three-dimensional structure of PulN. **Section E:** The purification of recombinant protein. **Section F:** The Lineweaver-Burk plot. (DOC 556 kb)

## Abbreviations

*pul*N: the pullulanase gene from *Paenibacillus polymyxa* Nws-pp2
Pul_N_: the pullulanase from *Paenibacillus polymyxa* Nws-pp2
LB: Luria Bertani medium
*kan*: Kanamycin
*amp*: Ampicillin
IPTG: Isopropyl-β-D-thiogalactopyranoside
SDS: Sodium dodecyl sulfate
SDS-PAGE: Sodium dodecyl sulfate-polyacrylamide gel electrophoresis
GHF: Glycosyl hydrolase family
DTT: Dithiothreitol
EDTA: Ethylene diamine tetraacetic acid
TLC: Thin layer chromatography
HPLC: High performance liquid chromatography
CCTCC: China Center for Type Culture Collection
DNS: 3,5-dinitrosalicylic acid reagent

## References

[1] Henrissat B (1991). A classification of glycosyl hydrolases based on aminoacidsequence similarities. Biochem J.

[2] Svensson B (1994). Protein engineering in the α-amylase family: catalytic mechanism, substrate specificity, and stability. Plant Mol Biol.

[3] Hii SL, Tau CL, Rosfarizan M, Ariff AB (2009). Characterization of Pullulanase Type II from *Bacillus cereus* H1.5. Am J Biochem Biotech.

[4] El-Shishtawy RM, Mohamed SA, Asiri AM, Gomaa AB, Ibrahim IH, Al-Talhi HA (2014). Solid fermentation of wheat bran for hydrolytic enzymes production and saccharification content by a local isolate *Bacillus megatherium*. BMC Biotechnol.

[5] Kłosowski G, Mikulski D, Czupryński B, Kotarska K (2010). Characterisation of fermentation of high-gravity maize mashes with the application of pullulanase, proteolytic enzymes and enzymes degrading non-starch polysaccharides. J Biosci Bioeng.

[6] Doman-Pytka M, Bardowski J (2004). Pullulan degrading enzymes of bacterial origin. Crit Rev Microbiol.

[7] Duffner F, Bertoldo C, Andersen JT, Wagner K, Antranikian G (2000). A new thermoactive pullulanase from *Desulfurococcus mucosus*: cloning, sequencing, purification, and characterization of the recombinant enzyme after expression in *Bacillus subtilis*. J Bacteriol.

[8] Rajaei S, Heidari R, Shahbani Zahiri H, Sharifzadeh S, Torktaz I, Akbari Noghabi K (2014). A novel cold-adapted pullulanase from *Exiguobacterium* sp. SH3: Production optimization, purification, and characterization. Starch-Starke.

[9] Kim CH, Choi HI, Lee DS (1993). Pullulanases of alkaline and broad pH range from a newly isolated alkalophilic *Bacillus* sp. S-1 and a *Micrococcus* sp. Y-1. J Ind Microbial Biot.

[10] Suzuki Y, Hatagaki K, Oda H (1991). A hyperthermostable pullulanase produced by an extreme thermophile, *Bacillus flavocaldarius* KP 1228, and evidence for the proline theory of increasing protein thermostability. Appl Microbial Biot.

[11] Ben Messaoud E, Ben Ammar Y, Mellouli L, Bejar S (2002). Thermostable pullulanase type I from new isolated *Bacillus thermoleovorans* US105: cloning, sequencing and expression of the gene in *E. coli*. Enzyme Microb Tech.

[12] Bertoldo C, Armbrecht M, Becker F, Schäfer T, Antranikian G, Liebl W (2004). Cloning, sequencing, and characterization of a heat-and alkali-stable type I pullulanase from *Anaerobranca gottschalkii*. Appl Environ Microb.

[13] Albertson GD, McHale RH, Gibbs MD, Bergquist PL (1997). Cloning and sequence of a type I pullulanase from an extremely thermophilic anaerobic bacterium, *Caldicellulosiruptor saccharolyticu*s. BBA-Gene Struct Expr.

[14] Li Y, Zhang L, Niu D, Wang Z, Shi G (2012). Cloning, expression, characterization, and biocatalytic investigation of a novel bacilli thermostable type I pullulanase from *Bacillus* sp. CICIM 263. J Agr Food Chem.

[15] Kang J, Park KM, Choi KH, Park CS, Kim GE, Kim D (2011). Molecular cloning and biochemical characterization of a heat-stable type I pullulanase from *Thermotoga neapolitana*. Enzyme Microb Tech.

[16] Bertoldo C, Duffner F, Jorgensen PL, Antranikian G (1999). Pullulanase type I from *Fervidobacterium pennavorans* Ven5: cloning, sequencing, and expression of the gene and biochemical characterization of the recombinant enzyme. Appl Environ Microbiol.

[17] Trincone A (2011). Marine biocatalysts: enzymatic features and applications. Mar Drugs.

[18] Lal S, Tabacchioni S (2009). Ecology and biotechnological potential of *Paenibacillus polymyxa*: a minireview. Indian J Microbiol.

[19] Wang SH, Yang Y, Zhang J, Sun J, Matsukawa S, Xie JL (2014). Characterization of abnZ2 (yxiA1) and abnZ3 (yxiA3) in *Paenibacillus polymyxa*, encoding two novel endo-1,5-α-l-arabinanases. Bioresources Bioprocessing.

[20] Castro GR, Santopietro LMD, Sineriz F (1993). Acid pullulanase from *Bacillus polymyxa* MIR-23. Appl Biochem Biotech.

[21] Kim HJ, Park JN, Kim HO, Shin DJ, Chin JE, Lee HHB (2002). Cloning and expression of a *Paenibacillus* sp neopullulanase gene in *Saccharomyces cerevisiae* producing *Schwanniomyces occidentalis* glucoamylase. J Microbiol Biotechn.

[22] Cai XH, Ma J, Wei DZ, Lin JP, Wei W (2014). Functional expression of a novel alkaline-adapted lipase of *Bacillus amyloliquefaciens* from stinky tofu brine and development of immobilized enzyme for biodiesel production. Antonie Van Leeuwenhoek.

[23] Wei W, Ma J, Guo S, Wei DZ (2014). A type I pullulanase of *Bacillus cereus* Nws-bc5 screening from stinkytofu brine: Functional expression in *Escherichia coli* and *Bacillus subtilis* and enzyme characterization. Process Biochem.

[24] Yamashita M, Matsumoto D, Murooka Y (1997). Amino acid residues specific for the catalytic action towards α-1,6-glucosidic linkages in *Klebsiella* pullulanase. J Ferment Bioeng.

[25] Wu S, Liu Y, Yan Q, Jiang ZQ (2014). Gene cloning, functional expression and characterisation of a novel glycogen branching enzyme from *Rhizomucor miehei* and its application in wheat breadmaking. Food Chem.

[26] Kusano S, Shiraishi T, Takahashi SI, Fujimoto D, Sakano Y (1989). Immobilization of *Bacillus acidopullulyticus* pullulanase and properties of the immobilized pullulanases. J Ferment Bioeng.

[27] Kunamneni A, Singh S (2006). Improved high thermal stability of pullulanase from a newly isolated thermophilic *Bacillus* sp. AN-7. Enzyme Microb Tech.

[28] Kuriki T, Park JH, Imanaka T (1990). Characteristics of thermostable pullulanase from *Bacillus stearothermophilus* and the nucleotide sequence of the gene. J Ferment Bioeng.

