# Supplementary material for: A novel cold-adapted type I pullulanase of Paenibacillus polymyxa Nws-pp2: in vivo functional expression and biochemical characterization of glucans hydrolyzates analysis
Source: BMC Biotechnol. 2015 Oct 19;15:96. doi: 10.1186/s12896-015-0215-z (PMC4615870; doi:10.1186/s12896-015-0215-z)
Supplement: Additional file 1: — Section A: The schematic representation of type I pullulanase. Section B: The bacterial strains, plasmids, chemicals and culture conditions. Section C: Pullulanase gene manipulation/propagation. Section D: The three-dimensional structure of PulN. Section E: The purification of recombinant protein. Section F: The Lineweaver-Burk plot. (DOC 556 kb) [file 12896_2015_215_MOESM1_ESM.doc]

**A novel cold-adapted type I pullulanase of *Paenibacillus polymyxa* Nws-pp2:** **in vivo functional expression and** **biochemical** **characterization of glucans hydrolyzates analysis**

Wei Wei*, Jing Ma, Si-Qi Chen, Xiang-Hai Cai, Dong-Zhi Wei*

State Key Laboratory of Bioreactor Engineering, Newworld Institute of Biotechnology, East China University of Science and Technology, Shanghai 200237, People’s Republic of China

* Corresponding author:

Wei Wei: Newworld Institute of Biotechnology, State Key Laboratory of Bioreactor Engineering, East China University of Science and Technology, 130 Meilong Road, 200237, Shanghai, China. Tel: +86-21-64251803, Fax: +86-21-64251803, Email: [lyuwei-1982@hotmail.com](mailto:lyuwei-1982@hotmail.com)

**Section A: The schematic representation of type I pullulanase**

**Section B: The bacterial strains, plasmids, chemicals and culture conditions**

**Section C: Pullulanase gene manipulation/propagation**

**Section D: The three-dimensional structure of Pul_N_**

**Section E: The puriﬁcation of recombinant protein**

**Section F: The Lineweaver-Burk plot**

**Section G: Nucleotide sequence accession in GeneBank**

**Section A: The schematic representation of type I pullulanase**

**
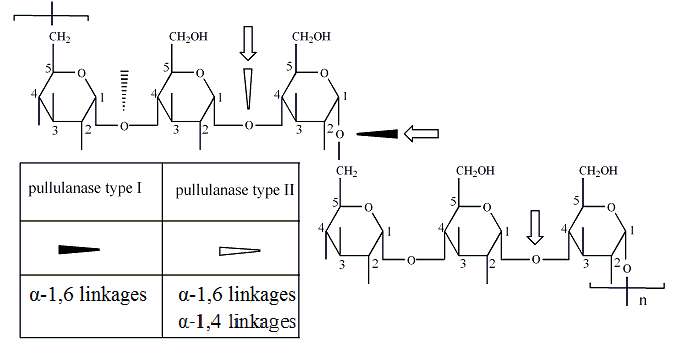
**

**Fig. S1** The schematic representation of type I pullulanase

**Section B: The bacterial strains, plasmids, chemicals and culture conditions**

**Table S1**

The bacterial strains and plasmids used in this study.

| Strains and plasmids | Relevant features | Source or reference |
| --- | --- | --- |
| **Strain** |  |  |
| *E. coli* DH5α | F¯ψ80lacZ∆M15∆(lacZYA-argF) U169 endA1 recA1 hsdR17 (rk¯,mk¯)supE44λ¯thi-1 gyrA96 relA1 phoA | BRL |
| *E. Coli* BL21(DE3) | F-, ompT, hsdS (rBB-mB), gal, dcm (DE3) | BRL |
| *Paenibacillus polymyxa* Nws-pp2 | wild strain screened from soil of fruit market garbage dump | CCTCC AB 2013352 |
| BL21-pET-28-*pul*N BL21-pET-32-*pul*N BL21-pET-42-*pul*N  **Plasmid** | BL21(DE3) harboring pET-28-*pul*N recombinant vector; Amp^r^  BL21(DE3) harboring pET-32-*pul*N recombinant vector; Kan^r^  BL21(DE3) harboring pET-42-*pul*N recombinant vectors; Kan^r^ | This study  This study  This study |
| pMD19-T | Cloning vector; Amp^r^ | Takara |
| pET-28a-c(+) | *E.coli* expression vector; Kan^r^ | Novagen |
| pET-28-*pul*N | pET-28a based vector, carrying mature pullulanase gene; Kan^r^ | This study |
| pET-32a-c(+) | *E.coli* expression vector; Amp^r^ | Novagen |
| pET-32-*pul*N | pET-32a based vector, carrying mature pullulanase gene; Amp^r^ | This study |
| pET-42a-c(+) | *E.coli* expression vector; Kan^r^ | Novagen |
| pET-42a-*pul*N | pET-42a based vector, carrying mature pullulanase gene; Kan^r^ | This study |

PrimeSTAR HS DNA Polymerase, T4 DNA ligase, DNA marker and restriction enzymes were purchased from TaKaRa Biotechnology Corporation (Otsu, Japan). Protein marker was purchased from MBI Fermentas (Vilnius, Lithuania). Isopropyl-β-D-thiogalactopyranoside (IPTG), ampicillin and kanamycin were purchased from Amresco (Shanghai Genebase Co., Ltd, China). DNA Mini kit and Plasmid Mini Prepare kit were purchased from Axygen Biosciences (Union City, CA, USA). The substrate pullulan, starch, amylose, amylopectin, glycogen and markers (glucose, maltose, maltotriose) were purchased from Sigma-aldrich Chemical Company (ST. Louis, MO, USA). Other chemicals were obtained commercially and were of the reagent grade.

Luria Bertani (LB) medium (bacto-tryptone 10.0 g/l, yeast extract 5 g/l, NaCl 10.0 g/l) was used for *E. coli* propagation with appropriate antibiotic. Yeast Peptone Dextrose (YPD) medium (bacto-tryptone 20.0 g/l, yeast extract 10.0 g/l, dextrose 20.0 g/l) was used for strain Nws-pp2 propagation. Pullulanase screening culture medium (pullulan 3.0 g/l, bacto-tryptone 5.0 g/l, KH_2_PO_4_ 0.5 g/l, MgSO_4_·7H_2_O 0.1 g/l, agar 20.0 g/l) was used for strain screening. Kanamycin (*kan*) was used at 50 μg/ml, ampicillin (*amp*) was used at 100 μg/ml, IPTG at 0.1 mM.

**Section C: Pullulanase gene manipulation/propagation**


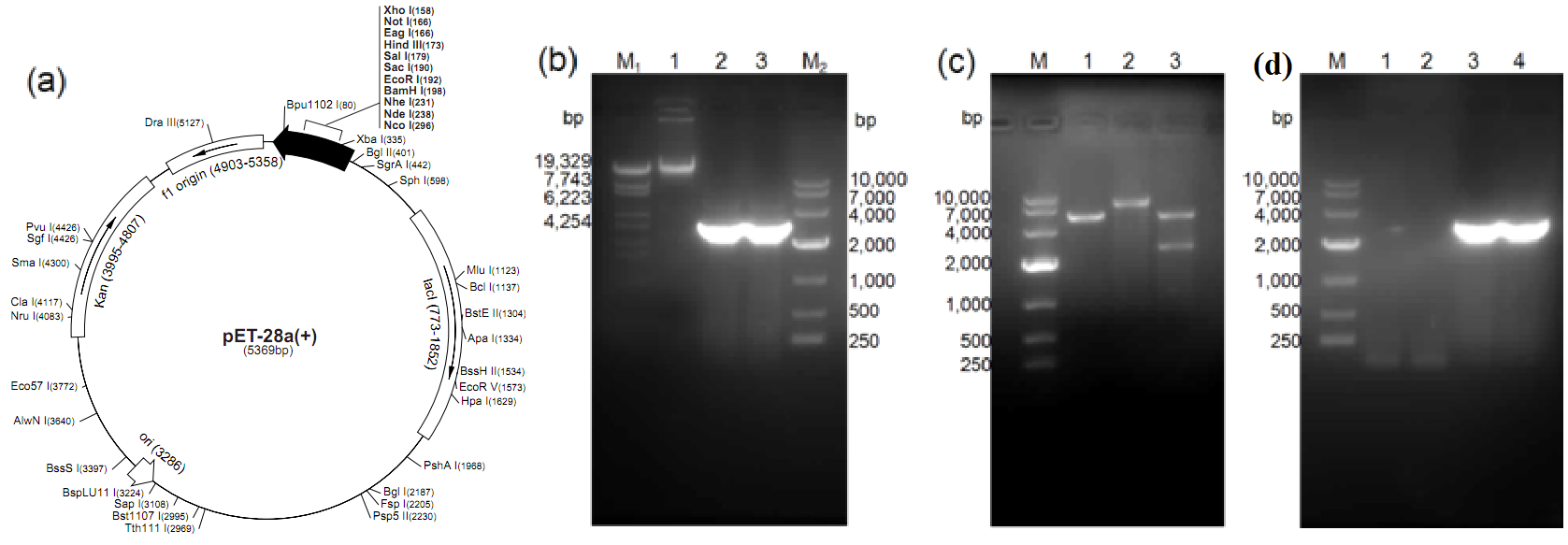


**Fig. S2** Result of gene cloning and verification of the recombinant plasmid (use BL21-pET-28-*pul*N as the example). (a) Empty pET28a plasmid. (b) Results of genome extraction and *pul*N gene choing. Lanes M: standard molecular weight markers, lanes 1:chromosomal DNA of *Paenibacillus polymyxa* Nws-pp2, lanes 2, 3: gene choing of *pul*N (about 2.5 kb). (c) Enzymatic verification of pET28a-*pul*N. Lanes M: standard molecular weight markers, lanes 1: pET28a digested by *Bam*HI, lanes 2: pET28a-*pul*N digested by *Bam*HI, lanes 3: pET28a-*pul*N digested by *Bam*HI and *Hind*III. (d) PCR verification of pET28a-*pul*N. Lanse M: standard molecular weight markers, lanes 1,2: PCR fragment of *E. coli* BL21(DE3)/pET28a, lanse 3,4: PCR fragment of *E. coli* BL21(DE3)/pET28a-*pul*N.

**Section D: The three-dimensional structure of Pul_N_**

**
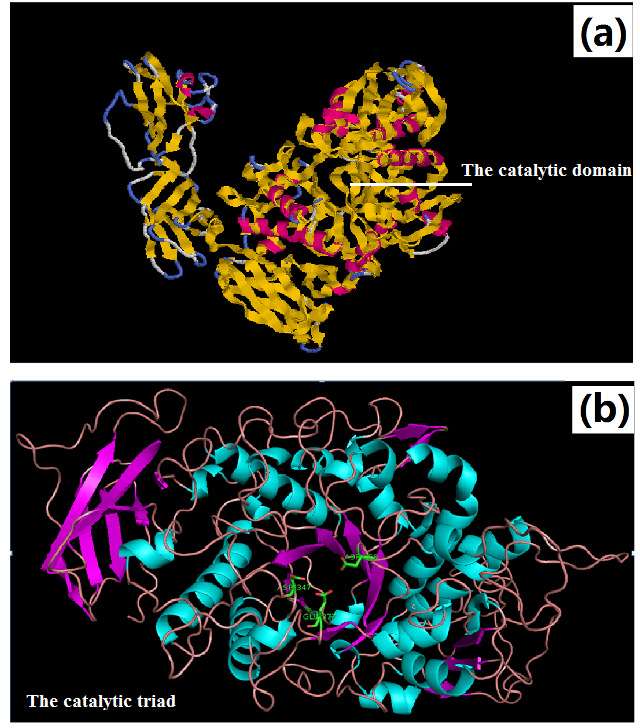
**

**Fig. S3** The three-dimensional structure of this enzyme was predicted by the SWISS-MODEL server. (a) The whole three-dimensional structure. (b) The catalytic domain of (α/β)_8_ structure. The catalytic triad Asp^347^, Glu^377^, and Asp^459^ residues were seen in the regions (in green).

**Section E: The puriﬁcation of recombinant protein**

After lysed by sonication, the crude enzyme was passed through a 0.22 μm filter and then applied to a Ni-NTA sperflow column (1 ml, Qiagen). After equilibrated with the lysis buffer (50 mM NaH_2_PO_4_, 300 mM NaCl, 10 mM imidazole, pH 8.0), the column was subsequently washed with 10 ml of wash buffer (50 mM NaH_2_PO_4_, 300 mM NaCl, 20 mM imidazole, pH 8.0) to remove the impurity protein. The fusion protein was eluted with the elution buffer (50 mM NaH_2_PO_4_, 300 mM NaCl, 100 mM imidazole, pH 8.0). The eluted protein was desalted and concentrated by ultrafiltration using a 50 ml Amicon Ultra Centrifugal Filter Device with a molecular weight cut-off of 10 kDa (Millipore, USA). The purified enzyme was resuspended in sodium phosphate buffer (pH 7.0) containing 20% glycerol and stored at -40^o^C. The crude extract and the pure enzyme were analyzed by SDS-PAGE. All purification steps were carried out at 4^o^C. Protein concentration was determined by the Bradford method with bovine serum albumin (BSA) as the standard.

**Section F: The Lineweaver-Burk plot**


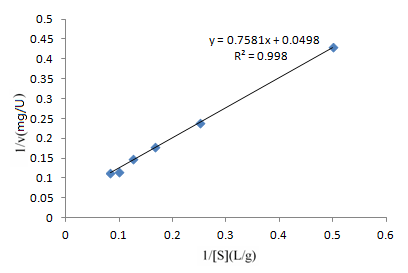


**Fig. S4** Lineweaver-Burk plot for PulN. Enzyme was incubated with pullulan at 35 °C and pH 6.0 for 10 min. The reciprocals of rate of substrate hydrolysis (1/V) were plotted against the reciprocals of the substrate concentrations (1/[S]), and the *Km* values were determined by ﬁtting the resulting data.

**Section G: Nucleotide sequence accession in GeneBank**

LOCUS KJ740392 2532 bp DNA linear BCT 15-JUL-2014

DEFINITION Paenibacillus polymyxa strain Nws-pp2 type I pullulanase gene,

complete cds.

ACCESSION KJ740392

VERSION KJ740392.1 GI:663081758

KEYWORDS .

SOURCE Paenibacillus polymyxa

ORGANISM [Paenibacillus polymyxa](http://www.ncbi.nlm.nih.gov/Taxonomy/Browser/wwwtax.cgi?id=1406)

Bacteria; Firmicutes; Bacilli; Bacillales; Paenibacillaceae;

Paenibacillus.

REFERENCE 1 (bases 1 to 2532)

AUTHORS Ma,J., Wei,W. and Cai,H.X.

TITLE Pullulanase Type I from Paenibacillus polymyxa Nws-pp2: Cloning and

Expression of the Gene, and Biochemical Characterization of the

Recombinant Enzyme

JOURNAL Unpublished

REFERENCE 2 (bases 1 to 2532)

AUTHORS Ma,J., Wei,W. and Cai,H.X.

TITLE Direct Submission

JOURNAL Submitted (21-APR-2014) East China University of Science and

Technology, Newworld Institude of Biotechnology, 130 Meilong Road,

Shanghai 200237, China

COMMENT ##Assembly-Data-START##

Sequencing Technology :: Sanger dideoxy sequencing

##Assembly-Data-END##

FEATURES Location/Qualifiers

source 1..2532

/organism="Paenibacillus polymyxa"

/mol_type="genomic DNA"

/strain="Nws-pp2"

/isolation_source="garbage soil"

/db_xref="taxon:[1406](http://www.ncbi.nlm.nih.gov/Taxonomy/Browser/wwwtax.cgi?id=1406)"

/collection_date="15-Feb-2012"

/PCR_primers="fwd_name: pp-u, fwd_seq: atgtctgatttcaatca,

rev_name: pp-d, rev_seq: ctatcccgctcgatcat"

[CDS](http://www.ncbi.nlm.nih.gov/nuccore/663081758?from=1&to=2532&sat=4&sat_key=116336304) 1..2532

/EC_number="[3.2.1.41](http://www.expasy.org/enzyme/3.2.1.41)"

/function="hydrolyzes alpha-1,6 glycosidic linkages in

pullulan or branched oligosaccharides forming maltotriose

or linear oligomers"

/codon_start=1

/transl_table=[11](http://www.ncbi.nlm.nih.gov/Taxonomy/Utils/wprintgc.cgi?mode=c#SG11)

/product="type I pullulanase"

/protein_id="[AIE88189.1](http://www.ncbi.nlm.nih.gov/protein/663081759)"

/db_xref="GI:663081759"

/translation="MSDFNQLKGLEHTAYPIYEGYDLGLTYTPAGSKFKVWAPTAQQV

HIALYSDAGVYDQEGIVQEHGGGQEFLMSRDDQGVWSIELEGDWNRYYYMYRLEWVDH

TIHYAADPYARAVSANGQRTAIIDLDQTNPDGWEQDEGPVLERATDAIIYELHVRDFS

MDPHFNTGIKDLSAAQGRFAAFTYTGLTDSEGNRIGLDHLLELGITHVHLLPVADFHT

VNDFSELGTEYNWGYDPQHYNVPEGSYSSNPADPEARIRELKLLVQSLHAAGIGVIMD

VVYNHTYSVEKGPFEPVVPGYYYRTDEQGKLTNGSGVGNEVATERPMVRKYIKDSLRY

WAEEYHIDGFRFDLMALIDTPTVEQLTRELRSEIRPDLLIYGEPWTGGESPQPLLTLK

GTQRDKGFAVFNDNYRSAIKGDSDGTGSGFATGAENQEERVLQGAFGAIYDFTAQPSE

TVNYVTAHDNLNLWDKILTVRHLREHCRFPQWEQGNPKDGRTAEQAVAEADPYQEMDE

SNLLENETVRKSLLANGLVLTSQGIPFIHAGDELLRSKYGDHNSYRSSDVVNAIRWNH

KARFRPVFDYYKGLIALRRSHPAFRIDQRELVEQHMEVLQSNGHVVAFALRHHANGDA

WNHIVVIYNGSDTEQTISLPAESDRWHVVVDAHGAGNETRYEVVGHRVTVSRWSMMVL

YDQDGPPQASFLTEHDHQANEQVDNKTKGLDEGTNQEIAGTAMTDAQLGNDREVIPFR

TIELIYERADRQYSGWNVWVWGTGYRDGHVEFQDLDDGRAIARIRVAPDVQRIGYIVR

LNHWDAKDVEADRYIDVDLTQSVMQVLIHSGREEYLLLVNDDRAG"

ORIGIN

1 atgtctgatt tcaatcagct caagggactt gagcacacag catatcccat atatgaaggc

61 tatgatttgg gtcttactta tacgcctgcg ggcagcaaat ttaaggtatg ggcaccgact

121 gcacagcagg ttcatatagc gttgtatagc gacgcagggg tgtacgatca ggaaggaatc

181 gttcaggagc acggtggtgg tcaggaattt ctaatgagcc gtgatgatca aggtgtatgg

241 tctatcgaac tggaaggcga ctggaacaga tattattaca tgtacaggct ggagtgggtg

301 gaccacacca ttcattatgc tgccgatcca tatgccagag cggtatcagc caacgggcaa

361 cgtacggcta tcattgattt ggatcaaacc aacccggatg gatgggaaca ggatgagggg

421 cctgtacttg agcgtgcgac agatgccatt atctatgagc ttcatgtgcg tgatttttcg

481 atggaccccc attttaatac gggtataaaa gatttatctg ccgctcaggg gcggtttgca

541 gcctttacgt atacgggatt aacggattcc gagggtaacc gtatcggact ggatcatttg

601 ctggagctgg gcattacaca tgttcatttg cttcccgtgg ctgactttca tacggtgaat

661 gacttttcgg aattaggaac tgaatataac tggggctatg atccgcagca ctataatgtg

721 ccggaggggt cctactcttc caatccagct gatccggagg cgcgtatacg cgagttgaaa

781 ttgctggtac agtctctgca tgcggccggc atcggtgtca ttatggacgt cgtgtacaat

841 catacgtatt cggtggaaaa gggtccgttt gagccagttg taccaggata ttattaccgt

901 acagatgagc aagggaagct caccaatgga tcaggtgtag gtaatgaggt ggcaaccgaa

961 cgtccgatgg tgcgcaagta tatcaaggat tcactacgct attgggcaga ggaatatcac

1021 atagatggct tccggtttga tttgatggcg ttgattgata caccgactgt agagcagctt

1081 actcgagaac ttcgctctga gatacgtccc gatctgctga tctatggtga gccttggaca

1141 ggaggagagt cccctcagcc tttgcttaca ttaaaaggaa cgcagcggga taagggtttt

1201 gccgttttta atgacaatta ccgttctgcc attaagggag atagcgacgg tacaggaagc

1261 gggtttgcta cgggtgcgga aaatcaggag gaacgggtac ttcaaggtgc ttttggagct

1321 atttatgatt tcacagctca gccatcagag accgtcaatt atgtaacagc ccacgataat

1381 ctcaatttgt gggataaaat tttgacggtg agacatttgc gggagcattg tcgttttccg

1441 caatgggagc aggggaaccc caaagatgga aggacagccg aacaagccgt agccgaagca

1501 gacccgtatc aggagatgga tgaaagtaac ctacttgaaa acgaaactgt acgtaagtcc

1561 ctgttagcta acggattggt tctgacatcg caaggcattc cttttatcca tgcaggggat

1621 gaactgctgc gttccaaata tggagaccat aacagctacc gaagctctga tgtggtgaac

1681 gccatccgtt ggaatcacaa ggcgaggttt cgacctgtgt ttgattacta caaaggctta

1741 attgctttgc ggcgcagtca cccggcattc cgtatagatc agcgtgagct ggtggagcag

1801 catatggaag tcctacaaag caatgggcat gtggtcgctt ttgccttgag gcatcatgct

1861 aacggcgatg cctggaatca tattgtggtg atttataacg gttcggatac agaacagacc

1921 atatcattgc ctgcggaatc ggaccgttgg catgtggtcg tggatgcaca cggggccggg

1981 aatgagacac ggtatgaggt ggtaggccat cgtgtgacgg tatcgcgctg gtcgatgatg

2041 gtgttgtacg atcaggatgg acctccccaa gcctcctttt tgacagagca tgatcatcaa

2101 gctaacgagc aagtggataa caaaacgaaa ggcttggatg aggggacaaa tcaagagatt

2161 gcaggtacag caatgacaga tgcccagcta ggaaacgata gagaggttat tccattccgc

2221 acaattgaat tgatctacga gcgtgcagac cggcaatact ctggctggaa tgtgtgggta

2281 tggggcactg ggtaccgtga cggacatgtt gaatttcaag atttagatga cggccgggca

2341 atagcccgca ttcgggtggc tcctgatgta cagcgaattg gctacattgt ccgcctgaat

2401 cactgggatg ccaaggatgt ggaggcagat cgatatattg atgtagatct gacccagtct

2461 gtgatgcagg tgttgattca tagtggcaga gaggagtatt tattactggt taacgatgat

2521 cgagcgggat ag

//
